# A gastric and pleural metastasis of occult breast lobular cancer in an elderly female and radiomics assisted analysis of a rare metastasis

**DOI:** 10.3389/fonc.2026.1703374

**Published:** 2026-02-06

**Authors:** Yuxin Xie, Qitao Gou, Wenjun Wu, Shuang Zhao, Libo Yang, Qiheng Gou

**Affiliations:** 1 Department of Medical Oncology, Cancer Center, West China Hospital, Sichuan University, Chengdu, China; 2 Breast Disease Center, West China Hospital, Sichuan University, Chengdu, China; 3 Department of Radiology, Union Hospital, Tongji Medical College, Huazhong University of Science and Technology, Wuhan, China; 4 Department of Radiology, West China Hospital, Sichuan University, Chengdu, China; 5 Department of Pathology, West China Hospital, Sichuan University, Chengdu, China; 6 Department of Radiation Oncology and Department of Head & Neck Oncology, Cancer Center, West China Hospital, Sichuan University, Chengdu, China

**Keywords:** gastric metastasis, immunohistochemistry, occult breast lobular carcinoma, pleura metastasis, radiomics analysis

## Abstract

**Background:**

Metastatic breast cancer involving the gastrointestinal tract is rare, particularly in lobular carcinoma, often emerging later in disease progression. Occult primary breast tumors are exceptionally uncommon. This study reports the first case of occult breast lobular carcinoma (OBLC) with concurrent gastric and pleural metastases in a 65-year-old female.

**Methods:**

A multidisciplinary diagnostic approach integrated histopathological and radiomic analyses. Immunohistochemical (IHC) profiles of axillary lymph node and gastric lesions were compared. Chest enhanced computed tomography (CT)-based radiomics quantified tumor texture features across five time points (T1-T5: pre- and post-treatment). The patient received aromatase inhibitors (AIs) combined with CDK4/6i as first-line treatment and chemotherapy as second-line treatment.

**Results:**

Immunohistochemistry confirmed consistent biomarker expression across metastatic sites, including ER positive, PR negative, HER2 negative and GATA3 positive. However, S100, SALL4, Syn, CDX2 and CgA were all expressed negatively in gastric metastatic lesions. Radiomics revealed progressive tumor brightness peaking at T3 (pre-treatment), followed by significant attenuation post-treatment (T4-T5). The ngtdm_Strength parameter increased markedly at T4-T5, compared to T1-T3, reflecting altered tumor vascularity after therapy. After two lines of treatment, the patient has survived for 24 months.

**Conclusions:**

This case highlights OBLC’s diagnostic complexity and underscores the role of radiomics in tracking metastatic evolution. Coordinated IHC and CT-based texture analysis aided lesion characterization and treatment monitoring for managing gastric metastases in OBLC.

## Background

Breast cancer is the most common malignant cancer and the second principal contributor to cancer-associated mortality in females (1). Invasive lobular carcinoma (ILC) is the second most common type of breast cancer, accounting for 5–15% of all breast cancer cases. While ILC typically metastasizes to bone, liver, and lungs, its predilection for gastrointestinal (GI) tract involvement - particularly gastric metastasis (6-18% of GI metastases) - warrants special attention given the diagnostic challenges it poses (2, 3).

The diagnostic complexity escalates in cases of occult breast carcinoma (OBC), representing 0.3-1% of breast malignancies (4). Defined by initial presentation with axillary lymph node metastases without identifiable primary breast lesions, OBC’s metastatic patterns remain undercharacterized. Notably, existing evidence suggests comparable prognosis between OBC and typical breast cancer when matched for nodal involvement, underscoring the critical need for accurate metastasis detection (5, 6).

Current diagnostic paradigms face limitations in detecting rare metastases, particularly in cases with ambiguous imaging presentations. Radiomics emerges as a transformative solution, enabling quantitative extraction of high-throughput image features from routine CT examination, which can reflect the biological information of the tumor and assist in making clinical diagnosis and treatment decisions (7). This computational approach has demonstrated success in characterizing various metastases (8, 9). However, its application in diagnosing GI metastases from breast cancer remains largely unexplored.

Herein, we report a postmenopausal female who was found to have a metastatic gastric lesion along with pleural and lymph node metastases from occult breast lobular carcinoma (OBLC). This report attempts to show the diagnosis of OBLC’s atypical metastasis patterns, demonstrate the implementation of CT-based radiomics for differentiating gastric metastasis from primary malignancies, and propose an integrative diagnostic workflow combining histopathological and radiomic analyses. The case underscores radiomics’ potential to augment conventional diagnostic modalities in managing metastatic breast cancer.

## Case presentation

**Figure 1:**
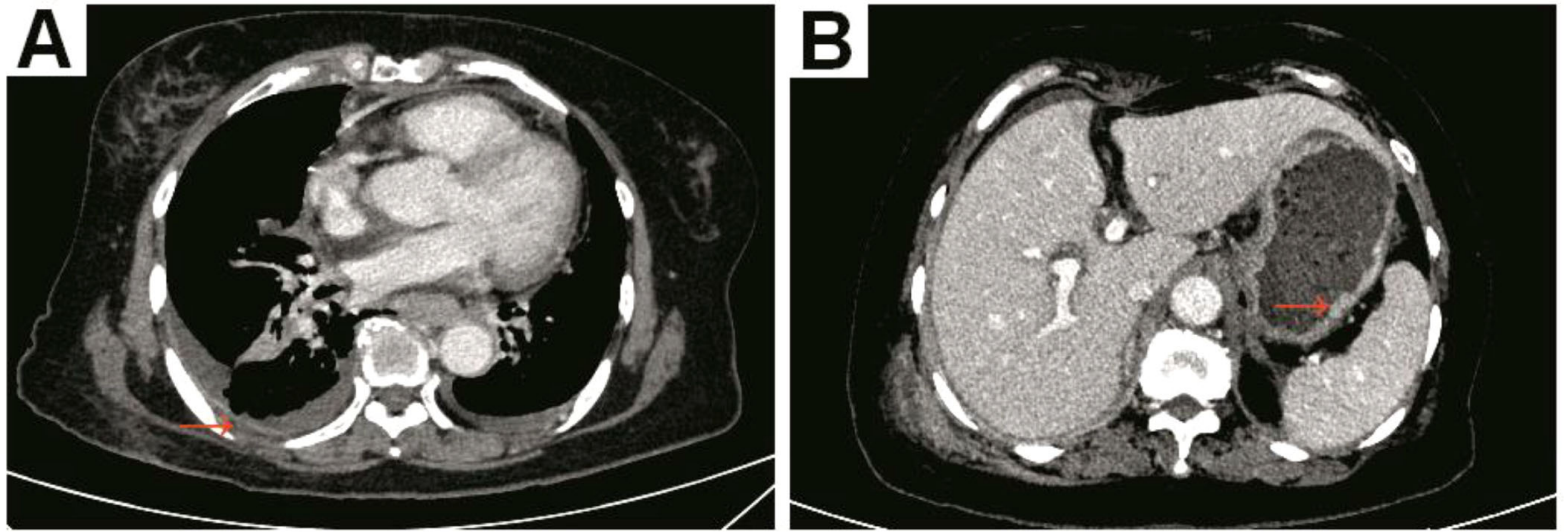
A chest enhanced computed tomography (CT) showed thickening of the right lateral parietal pleura (red arrow) with a small amount of pleural effusion **(A)**; an abdominal enhanced CT revealed findings consistent with gastric wall thickening and indistinct layers of the dorsal wall of the gastric body, accompanied by marked enhancement of the gastric mucosa (red arrow) **(B)**.

A 65-year-old postmenopausal female was found to have bilateral pleural effusion during a physical examination and presented with paradoxical weight loss (5 kg/month). However, the patient had no other symptoms and did not pay much attention to them. One month later, the patient experienced shortness of breath, accompanied by cough and dysphagia. She sought medical treatment at an emergency room in a local hospital, and pleurocentesis was performed to relieve her symptoms. Analysis of the pleural fluid revealed a lactate dehydrogenase (LDH) level of 202.00 U/L and an adenosine deaminase (ADA) level of 12.20 U/L. Despite receiving relevant symptomatic treatment, her symptoms did not improve. She was admitted to our hospital for further diagnosis and treatment. CT revealed not only bilateral pleural effusion but also thickening and swelling of the gastric and lower-middle segments of the esophageal mucosa at June 30, 2023 (**Figure 1**). Moreover, a mass (approximately 2.0 cm × 1.6 cm in size) was observed in the lower outer quadrant of the left breast. Additionally, the levels of the tumor markers carcinoembryonic antigen (CEA) and carbohydrate antigen 153 (CA153) were increased (12.20 ng/ml and >300.00 U/ml, respectively), while the level of CA199 was within the normal range (31.50 U/ml). Other examinations, such as abdominal ultrasound and biochemical tests, revealed no obvious abnormalities. She had a history of hypertension and schizophrenia and had been taking medication for a long time with good control.

**Figure 2:**
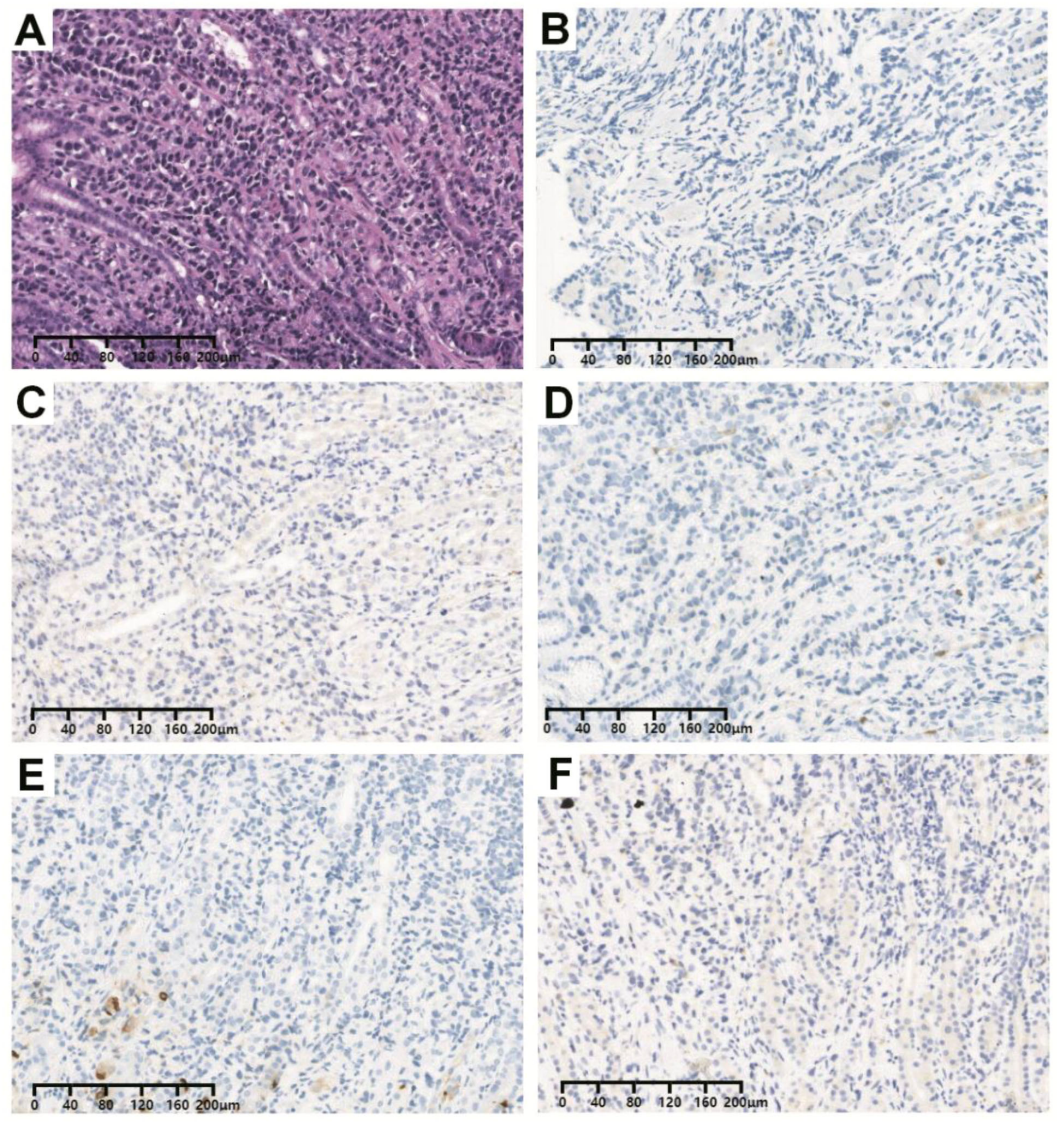
Representative H&E images **(A)** and immunohistochemistry images (IHC) of biopsy samples with **(B)** S100-negative expression, **(C)** SALL4-negative expression, **(D)** Syn-negative expression, **(E)** CDX2-negative expression and **(F)** CgA-negative expression. Abbreviations: SALL4, salt-like transcription factor 4; Syn, synuclein; CgA, chromogranin A.

**Figure 3:**
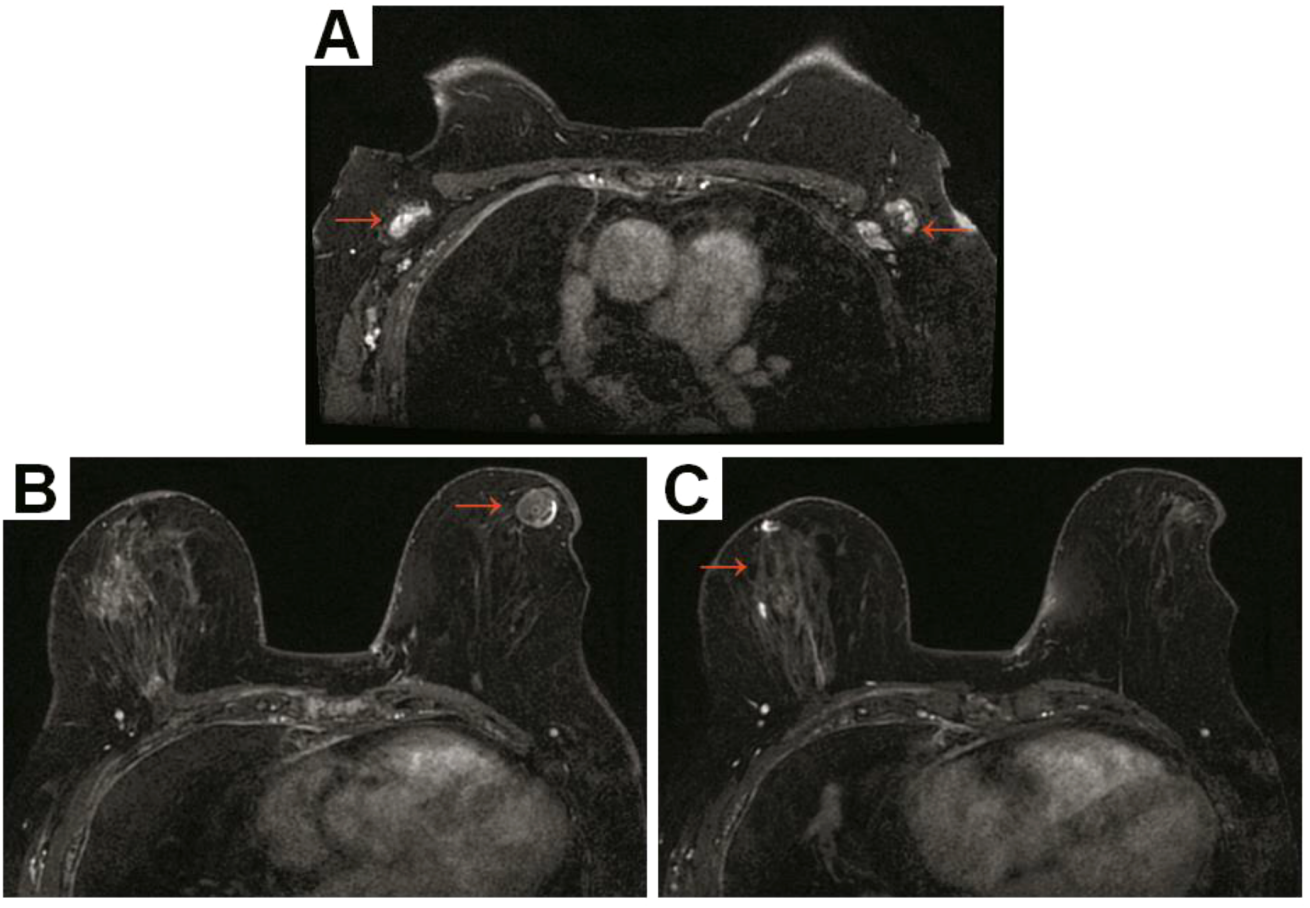
Breast magnetic resonance imaging revealed that bilateral axillary lymph nodes with enlargement and enhancement **(A)**, an oval shape lesion at 6 o’clock on the left breast, with heterogeneous enhancement and no diffusion limitation (BI-RADS category 4) **(B)** and a focal area of non-mass enhancement at 6 o’clock on the right breast with heterogeneous enhancement, no diffusion limitation (BI-RADS category 4) **(C)**.

**Figure 4:**
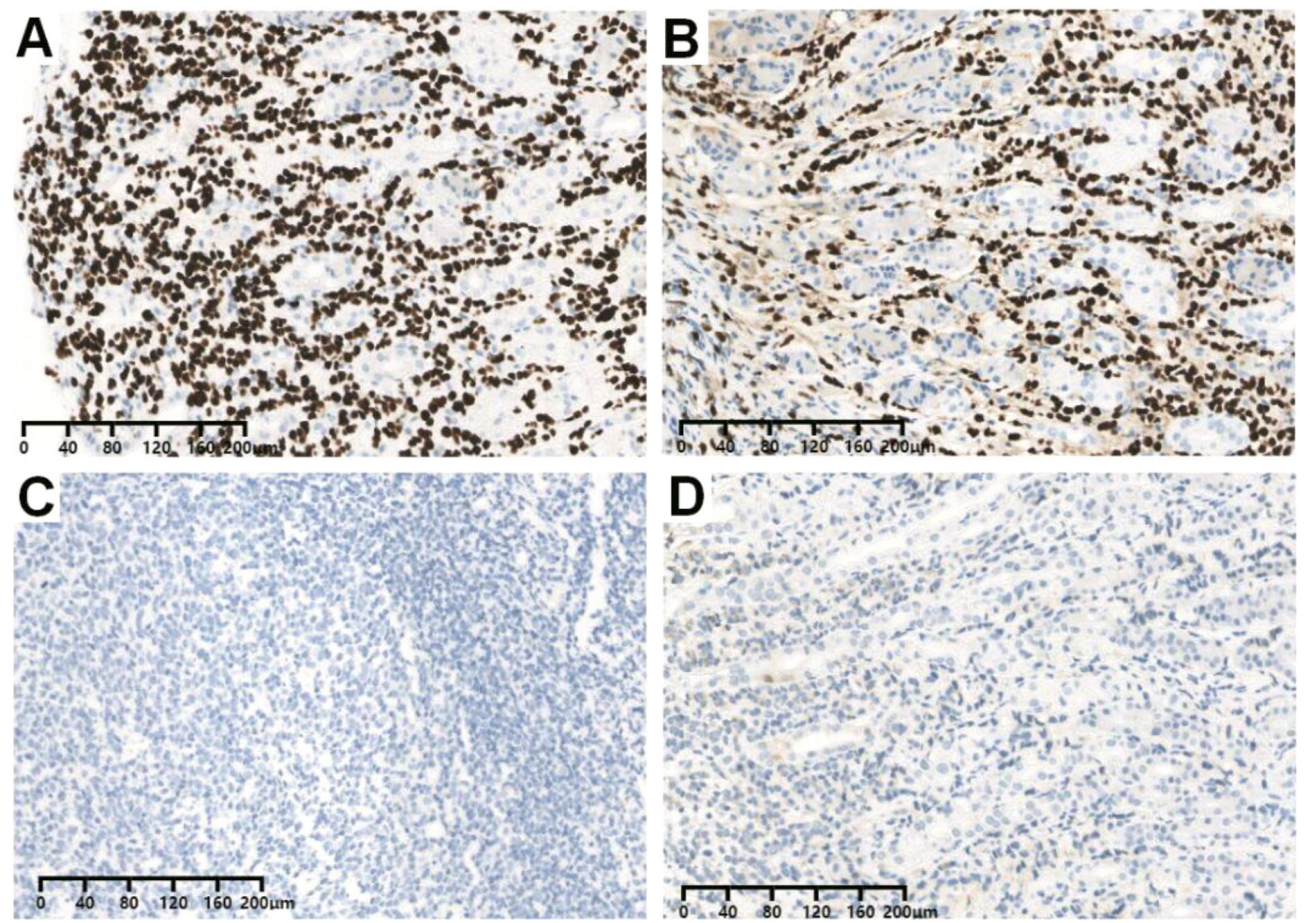
Representative immunohistochemistry images (IHC) of biopsy samples with **(A)** GATA3-positive expression, **(B)** ER-positive expression, **(C)** PR-negative expression, and **(D)** HER2-negative expression. Abbreviations: GATA3, GATA-binding protein 3; ER, estrogen receptor; PR, progesterone receptor; HER-2, human epidermal growth factor receptor 2.

Due to the abnormal features in the esophageal wall, an upper GI examination was subsequently performed. We did not find any abnormalities in the esophagus; however, gastroscopy revealed multiple areas of congestion and edema in the gastric mucosa. Four biopsies were taken, and the pathological report showed the presence of atypical cells in the greater curvature of the stomach. Pathological immunohistochemical (IHC) analysis revealed the following results: cytokeratin 7 (CK7) (+), CK20 (-), salt-like transcription factor 4 (SALL4) (-), S-100 (-), synaptophysin (Syn) (-), chromogranin A (CgA) (-), CDX2 (-), MLH1 (+), MSH2 (+), MSH6 (+), PMS2 (+) and Ki-67 (+, 10-20%) (**Figure 2**). The results supported the diagnosis of poorly differentiated adenocarcinoma (including signet ring cell carcinoma components). At the same time, the patient also underwent pleural biopsy through the pleural cavity. The pathological IHC results revealed the following: E-cadherin (-), CEA (+), CK7 (+), GATA-binding protein 3 (GATA3) (+), p120 (cytoplasmic +), estrogen receptor (ER) (+, 85%), progesterone receptor (PR) (+, 5%), human epidermal growth factor receptor 2 (HER-2) (0), special AT-rich sequence-binding protein 2 (SATB2) (+), thyroid transcription factor 1 (TTF-1) (-), CDX2 (-), CK20 (-), CR (-), Villin (-), WT1 (-), and Pax-8 (-). The results prompted us to further differentiate between primary lesions originating from breast lobular carcinoma and those originating from poorly differentiated adenocarcinoma of the digestive system. Thus, contrast-enhanced ultrasound of the breast was further performed, and the report showed the following: (1) a solid mass at 4 o’clock in the left breast (21 × 12 × 17 mm) without typical malignant signs, and the best imaging reporting and data system category was 4B (BI-RADS category 4B); (2) a 6 o’clock mass (46 × 19 mm) in the right breast was suspected to be a dermatofibrosarcoma protuberans (DFSP) (BI-RADS category 4B). Aspiration biopsy of the bilateral breast mass was recommended. Moreover, bilateral axillary lymph nodes were enlarged. In addition, breast magnetic resonance imaging (MRI) was also performed. In addition to bilateral axillary lymph node enlargement, MRI suggested non-mass-like enhancement in the central area of the right breast (BI-RADS category 4) and a mass in the area of the left breast areola (BI-RADS category 4) (**Figure 3**). To clarify the primary lesion, the bilateral breast masses and multiple enlarged lymph nodes of the patient were further diagnosed using ultrasound-guided core needle biopsy (US-CNB). The bilateral lymph node pathological IHC results were as follows: (1) GATA3 (+), GCDFP-15 (+), P63 (-), E-C (-), P120 (cytoplasmic +), β-catenin (-), ER (+, 90%), PR (-), Her-2 (1+), CK5/6 (-), CK (+), TTF-1 (-), and Ki-67 (+, ≤20%). The results revealed infiltrating lobular carcinoma in the axillary lymph nodes, whether from the breast or accessory breast. However, no tumor cells were found at 4 or 6 o’clock in the left or right breast, respectively. Based on the IHC and histopathological findings, our analysis focused on the expression profiles of GATA3, ER, PR, and HER-2 in the gastric biopsy specimens. Notably, the gastric mucosa, pleural lesions, and axillary lymph node metastases demonstrated consistent immunoprofiles across these markers (**Figure 4**). This immunohistochemical concordance strongly supports the conclusion that the initial gastric lesions identified in this patient represent metastatic carcinoma, which has been pathologically confirmed as originating from OBLC metastasis.

Following a comprehensive physical examination on August 3, 2023, the patient was treated with abemaciclib plus exemestane which was the first-line therapy for postmenopausal women with hormone receptor (HR)-positive, HER2-negative advanced breast cancer. Concurrent management of malignant pleural effusion (MPE) was achieved through intrathoracic infusions of bevacizumab and cisplatin administered monthly. This regimen yielded a progression-free survival (PFS) of 4.3 months. Serial imaging surveillance revealed disease progression on December 27, 2023, with CT demonstrating: increased pleural fluid accumulation; new chest wall soft tissue mass suggestive of metastatic involvement; progressive thickening of gastric wall, small intestinal wall, and rectal wall, consistent with carcinomatous peritonitis; multiple new osteolytic lesions indicative of widespread skeletal metastases. Thus, second-line therapy was initiated with albumin-bound paclitaxel combined with capecitabine. This regimen maintained disease stability for >15 months with acceptable tolerability. Bone-modifying therapy with denosumab (120 mg SC monthly) was concurrently administered for skeletal-related event (SRE) prophylaxis, demonstrating effective preservation of skeletal integrity throughout the treatment period.

### Radiomics methods

Five longitudinal contrast-enhanced chest CT time points were analyzed: T1 (Jun 30, 2023), T2 (Aug 3, 2023), T3 (Oct 27, 2023) – pre-treatment/early treatment; T4 (Dec 27, 2023), T5 (Feb 27, 2024) – post-progression/on second-line therapy. Regions of interest (ROIs) encompassing the thickened gastric wall from cardia to angle were manually contoured on portal venous phase images using 3D Slicer (v5.6.1). Radiomics features were extracted using the SlicerRadiomics plugin.

Given the visual and clinical distinction between the early/thickened phase (T1-T3) and the later/treated phase (T4-T5), a two-group comparative analysis was performed. Key radiomics features were selected based on their known biological correlates and relevance to treatment response monitoring. Their definitions and clinical interpretations are summarized in **Table 1**.

**Table 1:** Key radiomics features analyzed and their clinical interpretation.

Feature class	Feature name	Description	Biological/clinical correlation	Trend observed (T1-T3 vs T4-T5)
First-Order Statistics	90 Percentile	The voxel intensity value below which 90% of the ROI voxels lie. Reflects high-end brightness.	Related to tissue density and enhancement. High values may indicate vigorous contrast uptake (e.g., hypervascular tumor).	Peaked at T3, then significantly decreased at T4-T5.
Texture (NGTDM)	Strength	Reflects the intensity of grayscale differences between a voxel and its neighborhood. Measures local heterogeneity.	High values suggest coarse texture and pronounced local variation, which may decrease as tumor homogeneity changes post-therapy.	Markedly increased in T4-T5 compared to T1-T3.

### Radiomics analysis during the course of the disease

**Figure 5:**
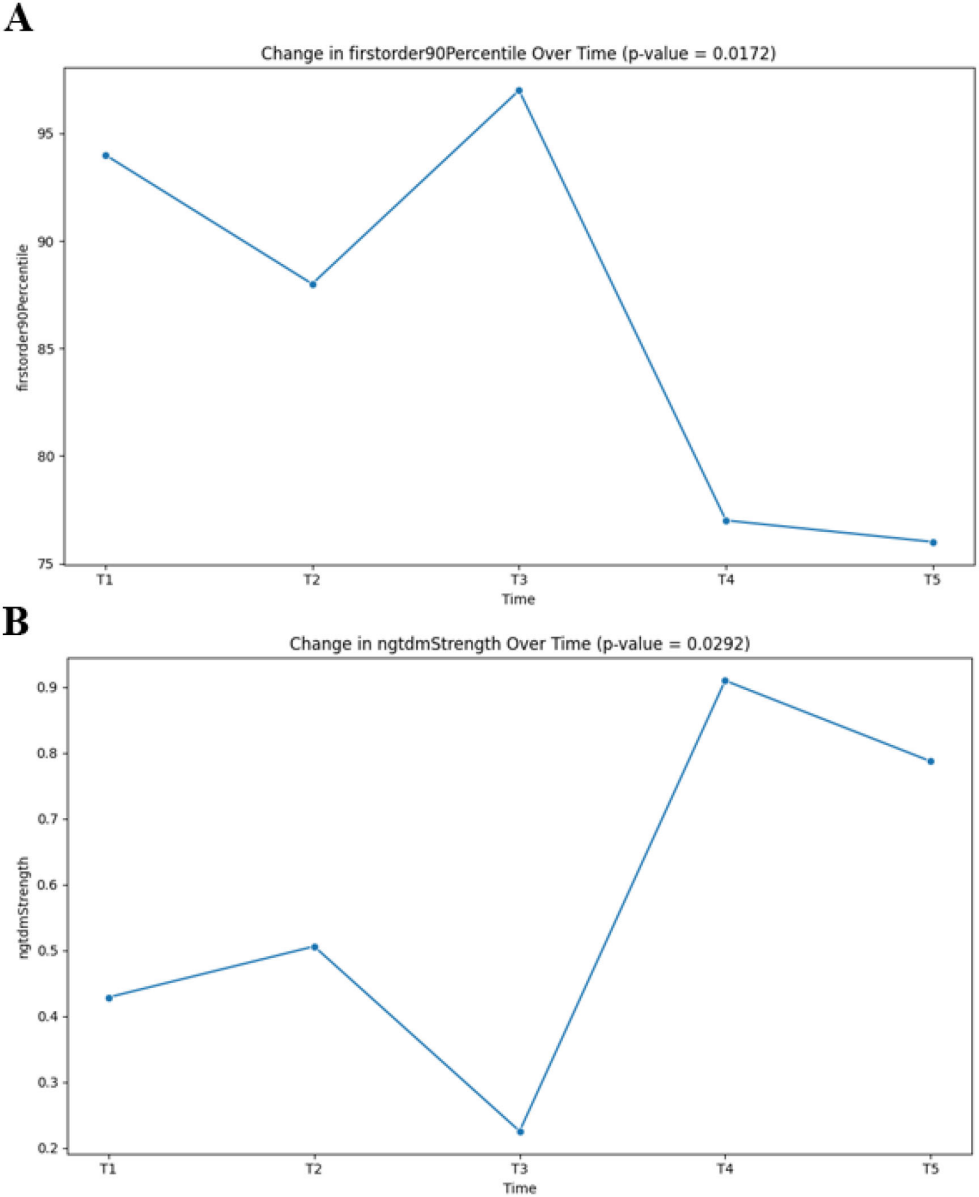
Radiomics analysis of patients’ previous course of illness. **(A)** Feature analysis of first-order 90 percentile. The y-axis represents the grayscale values corresponding to 90% of the voxels (pixel values) in the image grayscale histogram, and the x-axis represents the results of each chest enhanced CT scan (T1-T5). **(B)** Feature analysis of ngtdm_Strength. The y-axis represents the intensity of grayscale differences between a voxel and its neighborhood, while the x-axis represents the results of previous chest enhanced CT scans (T1-T5). Time point of chest enhanced CT scan. T1: June 30, 2023, T2: August 3, 2023, T3: October 27, 2023, T4: December 27, 2023, T5: February 7, 2024.

The first-order 90 Percentile feature, indicating high-intensity voxels, showed that tumor brightness increased during the pre-treatment/early treatment phase, peaking at T3. A significant decrease was observed at T4 and T5 (**Figure 5A**), correlating with the period following first-line therapy and potentially indicating reduced tumor vascularity and cellularity.

The texture feature ngtdm_Strength demonstrated lower values during T1-T3 but increased substantially at T4-T5 (**Figure 5B**). This suggests a shift in the local heterogeneity pattern of the gastric wall after disease progression and initiation of second-line therapy, possibly reflecting treatment-induced changes in the tumor microenvironment and restoration of more normal gastric wall architecture.

## Discussion

We present a rare case of OBLC with synchronous gastric and pleural metastases, highlighting the diagnostic challenges and therapeutic implications of this elusive malignancy. The patient initially manifested with bilateral pleural effusion, characterized by elevated LDH and decreased ADA levels in pleural fluid analysis, indicating malignant pleural effusion, which was subsequently confirmed by pathological biopsy. Contrast-enhanced CT demonstrated bilateral pleural effusion, diffuse gastric and esophageal mucosal thickening, and a suspicious left breast mass. Notably, serum tumor markers CEA and CA153 were significantly elevated. Comprehensive diagnostic workup included gastroscopic biopsy showing poorly differentiated adenocarcinoma on histopathology, while initial breast tissue biopsy remained inconclusive. However, axillary lymph node fine-needle aspiration cytology combined with pleural effusion IHC strongly suggested breast origin. Further pleural biopsy and molecular profiling confirmed metastatic OBLC. To date, this patient has received two lines of systemic therapy and maintains an overall survival exceeding 18 months since initial diagnosis. The patient underwent targeted therapy with abemaciclib and exemestane, supplemented by intrathoracic bevacizumab-cisplatin combination therapy, achieving PFS of 4.3 months prior to disease progression. Current treatment regimen includes 8 cycles of albumin-bound paclitaxel with capecitabine, complemented by denosumab for skeletal event prophylaxis.

A critical differential diagnosis was primary gastric signet-ring cell carcinoma versus metastatic lobular carcinoma. Both can present with diffuse infiltration and signet-ring morphology. Key IHC discriminators include: Primary Gastric Adenocarcinoma: Typically CK20+/CDX2+/GATA3- (intestinal type) or variably positive (diffuse type). Metastatic Breast Lobular Carcinoma: Typically CK7+/GATA3+/ER+/GCDFP-15+/E-cadherin-/CK20-/CDX2- (10). The concordant GATA3 and ER positivity across gastric, pleural, and nodal lesions in our patient definitively ruled out a primary gastric cancer and confirmed metastatic breast origin. This underscores the necessity of a broad IHC panel when faced with poorly differentiated adenocarcinoma in the stomach of a female patient.

OBLC, a subtype of OBC, typically presents with axillary lymphadenopathy or metastatic manifestations rather than primary breast lesions. The onset of OBC usually occurs at approximately 50 years of age, and its incidence among all types of breast cancer is approximately 0.3% to 1% (4, 6). Histologically, OBLC shares characteristics with ILC - the second most prevalent breast malignancy after invasive ductal carcinoma (IDC). ILC often presents with subtle thickening or fullness rather than a distinct lump, complicating early detection (10, 11). Patients with ILC often have a positive ER/PR status but a negative HER2 status (12, 13). Moreover, ILCs are characterized by the near-universal loss of the cell adhesion protein E-cadherin. This means that there is a greater likelihood of distant metastasis (10, 14). Advanced imaging modalities, particularly breast MRI, play a crucial role in detecting radiographically occult primaries associated with axillary metastases (15).

GI metastases in patients with OBLC are relatively uncommon (12–15). Most documented cases of metastatic OBLC are limited to case reports and small case series, underscoring its rarity. Autopsy studies suggest that gastric metastases represent the most frequent gastrointestinal involvement in advanced breast cancer (3). Different histological types of breast cancer exhibit distinct metastatic patterns. While both IDC and ILC commonly disseminate to the lung, liver, and bone, ILC exhibits a marked predilection for metastasis to the gastrointestinal tract, peritoneum, and gynecological organs (13, 16, 17). This may be attributed to the non-cohesive growth pattern and loss of E-cadherin characteristic of lobular carcinoma, facilitating diffuse infiltration of visceral sites. Our patient represents such a case of gastric metastasis originating from breast lobular carcinoma.

We systematically searched the PubMed, MEDLINE, Embase, and Google Scholar databases, identifying 5 case reports of OBLC with gastric metastasis through our inclusion criteria (**Table 2**) (7, 18–21). Notably, all cases were histologically confirmed as ILC, predominantly affecting middle-aged to elderly individuals. The most frequent presenting symptoms included weight loss, abdominal pain, fatigue, and diarrhea, with no significant family history correlation. Intriguingly, while integrated case analyses revealed no marked elevation of tumor markers in ILC patients, our case demonstrated prominent elevations in both CEA and CA15-3, suggesting potential biomarker variability in metastatic progression (20). Of particular diagnostic significance, the CK7+/CK20- immunophenotype observed in our patient coupled with GATA3 positivity and negative CDX2 expression, effectively excluded gastrointestinal origin while confirming breast primary. This immunoprofile aligns with established patterns where metastatic breast carcinomas typically express CK7/ER/PR/GCDFP-15 but lack CK20/CDX2 (17). Current evidence underscores the indispensable role of IHC in diagnosing OBLC related gastric metastases, particularly when biomarker discordance exists.

**Table 2:** Five patients with gastric metastases originating from occult breast carcinoma.

Reference	Age	Gender	Family history	Chief complain	Other site of metastatic	Histologic type	IHC	Treatment	Survival statue
Ciulla A et al., 2008 (18)	70	Female	N/A	Vomiting; weight loss	N/A	ILC	ER (+, 60%); PR (+, 40%); Her-2 (-); CK7 (+); CK20 (-); GCDFP15 (+)	Total gastrectomy and esophago-jejunostomy; hormone therapy	Death (10 month)
Wagner J et al., 2009 (22)	78	Female	N/A	Fatigue; decrease appetite; weight loss; diarrhea	Coecal	ILC	ER (+, 80%); PR (+, 10-15%); Mammoglobin (+, 30%); TTF-1 (-); CK 5/6 (-); CK 20(-); E-Cadherin (-).	Tamoxifen	Alive
Neal L et al., 2009 (23)	53	Female	Positive	Anorexic; fatigue; diarrhea	Colon; chest wall	ILC	ER (+, 90%); PR (+, 10%); CK7 (+); HER2 (-); CK20 (-); CD45 (-); CDX2 (-)	Hormone therapy	Alive
Zuhair AR et al., 2015 (19)	47	Female	N/A	Abdominal pain	N/A	ILC	ER (+, strong); PR (-); Her-2 (-)	Bilateral mastectomy; hormone therapy; chemotherapy	Alive
Pouptsis A et al., 2024 (11)	51	Female	Positive	Abdominal pain, weight loss and fatigue	Abdominal, bilateral ovarian, ascending colon, mesenterial and peritoneal.	ILC	CK7 (+); mammaglobin (+); GATA3 (+); GCDF15 (+); ER (+); PR (+); HER-2 (-); CK20 (-); CDX2 (-); PAX8 (-); WT1 (-)	Hormone therapy and CDK 4/6 inhibitor;	Alive

In view of the rare metastasis of breast cancer, the position of enhanced CT cannot be doubted, but the interpretation of its results is often based on the clinical experience of radiologists, which often needs to be combined with other different types of examinations to be determined (16). Imageomics can extract high-throughput image features of the focus to assist in the interpretation of the results, providing support for clinical diagnosis and treatment. For changes in grayscale values, first-order 90 Percentile can be used, which is also one of the first-order statistical features used to represent the grayscale values corresponding to 90% of the voxels (pixel values) in the image grayscale histogram. It can be used for evaluating brightness distribution, detecting and classifying lesions, and monitoring treatment effectiveness (17). In this case, as the diagnosis and treatment progressed, the brightness of the tumor area significantly decreased after T4 time point, which may be due to reduced blood supply, tumor reduction, and decreased enhancement components after treatment. This is also consistent with the abemaciclib plus exemestane time point. In addition, we often need to analyze the grayscale changes within and around the tumor area, and ngtdm_Strength is one of the features based on the neighbor gray-tone difference matrix (NGTDM), which measures the degree of grayscale changes in the image. It reflects the intensity of grayscale differences between a voxel and its neighborhood. It can be used for detecting tumor texture analysis, assessing tumor heterogeneity, and predicting treatment response. In this case, compared to the initial T1-T3 time points, the results at T4-T5 time points were higher. This may be due to the gradual restoration of normal gastric wall blood supply after treatment, resulting in a decrease in overall enhancement heterogeneity, which corresponds to the first-order 90 Percentile results. Therefore, this article provides two features in the imageomics of a rare breast cancer with gastric metastasis. Combining other features and clinical information, it can provide more comprehensive tumor evaluation and monitoring. Based on this example, the characteristics of CT radiomics in gastric metastasis mode can be explored, providing theoretical support for inferring diagnosis, efficacy evaluation, and prognosis through CT imaging.

This case demonstrates the adjunctive value of radiomics in managing rare metastases. The quantitative features (90 Percentile, ngtdm_Strength) provided objective measures of tumor phenotype evolution that complemented anatomical imaging. The observed trends—decreased brightness post-therapy and altered texture heterogeneity—align with expected biological responses (reduced enhancement) and structural changes. The translational implication lies in potentially integrating such radiomics signatures into future diagnostic support systems. For instance, in a patient with axillary nodal carcinoma of unknown primary and suspicious gastric thickening, a radiomics classifier trained on features differentiating primary vs. metastatic gastric lesions could prioritize breast origin in the differential. Furthermore, longitudinal radiomics could serve as a sensitive biomarker for early treatment response assessment, especially in lesions difficult to measure by RECIST.

Currently, there are no established clinical guidelines or expert consensus to direct the management of OBC with oligometastatic involvement (6). Previous studies showed that patients who were initially diagnosed with OBC without distant metastasis underwent mastectomy or resection of metastatic lesions (18, 19). While the National Comprehensive Cancer Network (NCCN) guidelines endorse mastectomy as the principal intervention for OBC, they notably lack specific recommendations for cases complicated by gastric oligometastases (4). From a systemic therapy perspective, ILC generally follows treatment paradigms similar to other histological subtypes. However, its characteristic chemoresistance necessitates alternative therapeutic strategies. Emerging evidence suggests that endocrine-based regimens incorporating CDK4/6 inhibitors demonstrate superior clinical responses and survival benefits in HR+/HER2- disease (20). The MONARCH 3 and MONARCH PLUS trials substantiate these findings, reporting significant improvement in PFS with continuous abemaciclib administration combined with AIs in postmenopausal women with advanced HR+/HER2- breast cancer (20, 21). However, this regimen achieved modest disease control with a PFS of 4.3 months in the case. Following rapid radiological progression evidenced by RECIST 1.1 criteria, second-line therapy was instituted using albumin-bound paclitaxel with capecitabine. Remarkably, this therapeutic transition resulted in sustained disease stabilization exceeding 15 months of ongoing PFS. The treatment course was generally well-tolerated, with grade 3 thrombocytopenia (CTCAE v5.0) representing the most significant adverse event, effectively managed through dose modification and supportive care.

However, This is a single-case report, which inherently limits the generalizability of the findings. The radiomics analysis is exploratory; the observed feature changes, while biologically plausible, require validation in larger cohorts. The lack of comprehensive molecular profiling (e.g., genomic sequencing) of the metastatic lesions precludes deeper insights into the driver mechanisms of this atypical spread.

## Conclusions

We present a rare case of gastric and pleural metastases from OBLC in a postmenopausal woman. IHC profiling of gastric biopsy specimens (e.g. GATA3+/ER+/CK7+) confirmed mammary origin despite inconclusive breast imaging. Unlike previous cases, the patient exhibited markedly elevated tumor biomarkers (CA153 and CEA), reflecting the invasiveness of tumors. We collected and analyzed relevant case reports, revealing that ILCs account for most gastric metastases in patients with OBC. We aim to provide a basis for clinical decision-making and encourage practitioners to publicize these rare carcinomas. Additionally, radiomics was used to analyze treatment-related tumor changes, offering a theoretical basis for diagnosis and efficacy evaluation of this rare metastasis. Overall, standardized biomarker assessment and advanced imaging analytics may optimize diagnostic precision and therapeutic outcomes in these rare yet clinically consequential metastases.

## Data Availability

The original contributions presented in the study are included in the article/aupplementary material. Further inquiries can be directed to the corresponding authors.
